# Cost effectiveness of a 21-valent pneumococcal conjugate vaccine in adults: A systematic review of economic evaluations

**DOI:** 10.14745/ccdr.v51i23a03

**Published:** 2025-02-12

**Authors:** Alison E Simmons, Raphael Ximenes, Gebremedhin B Gebretekle, Marina I Salvadori, Eva Wong, Ashleigh R Tuite

**Affiliations:** 1 Centre for Immunization Surveillance and Programs, Public Health Agency of Canada, Ottawa, ON; 2 Dalla Lana School of Public Health, University of Toronto, Toronto, ON; 3 Department of Pediatrics, McGill University, Montréal, QC

**Keywords:** 21-valent pneumococcal conjugate vaccine, pneumococcal disease, vaccination, cost-utility analysis, health economics

## Abstract

**Background:**

In July 2024, Health Canada authorized a 21-valent pneumococcal conjugate vaccine (Pneu-C-21) for use in adults.

**Objective:**

To conduct a systematic review of the cost-effectiveness of Pneu-C-21 for preventing pneumococcal disease in adults.

**Methods:**

We conducted a systematic search of the literature and National Immunization Technical Advisory Groups’ websites on July 3, 2024. We included economic evaluations that assessed Pneu-C-21 as a vaccination strategy among adults aged 18 years and older. Costs were adjusted to 2023 Canadian dollars.

**Results:**

We identified 10 studies in our search, five of which were summarized in our review. No economic evaluations were conducted in Canada. All economic evaluations used static cohort models and incorporated indirect effects from paediatric pneumococcal conjugate vaccination in primary or sensitivity analyses. Although incremental cost-effectiveness ratios were heterogeneous across included economic evaluations, overall, they qualitatively identified the same vaccination strategies as optimal within the given age and risk groups. Pneu-C-21 is likely to be cost-effective in adults aged 65 years and older and adults under the age of 65 years with specific high risk conditions.

**Conclusion:**

Pneu-C-21 is likely to be cost-effective in adults within specific age and risk groups. The applicability of the included economic evaluations to adults living in Canada is limited because the serotype-specific incidence of pneumococcal disease and the impact of indirect effects from pediatric vaccination varies by region and over time.

## Introduction

The bacterium *Streptococcus pneumoniae* is a significant cause of morbidity and mortality in Canada and worldwide ((1)). Of over 100 known serotypes of *S. pneumoniae* ((2)), 15 cause the majority of disease in Canada ((1)). The upper respiratory tracts of between 20%–60% of children and approximately 10% of healthy adults are colonized with *S. pneumoniae* ((3)). In rare cases, there is infection of a normally sterile site (e.g., blood, meninges), causing invasive pneumococcal disease (IPD).

There are a number of pneumococcal vaccines authorized for use in Canada, including the 15- and 20-valent pneumococcal conjugate vaccines (Pneu-C-15 and Pneu-C-20, respectively) and the 23-valent pneumococcal polysaccharide vaccine (Pneu-P-23) ((4)), that aim to protect vaccine recipients from severe disease caused by 15-, 20- or 23-valent *S. pneumoniae* serotypes. Canada’s National Advisory Committee on Immunization (NACI) currently recommends the use of Pneu-C-20 in adults at high risk of IPD, including adults aged 65 years and older and adults under 65 years of age with medical or social risk factors.

In July 2024, Health Canada approved a 21-valent pneumococcal conjugate vaccine (Pneu-C-21) for use in individuals aged 18 years and older ((5)). One month prior, in June 2024, the United States Advisory Committee on Immunization Practices recommended Pneu-C-21 as an option for adults aged 19 years and older who were recommended to receive Pneu-C-15 or Pneu-C-20 ((6)). Pneu-C-21 contains 10 unique non-cross-reactive serotypes (9N, 15A, 16F, 17F, 20A, 23A, 23B, 24F, 31 and 35B) compared to Pneu-C-20, and Pneu-C-20 contains nine unique serotypes not included in Pneu-C-21 (1, 4, 5, 6B, 9V, 14, 18C, 19F and 23F). Using established methodology to assess the benefit of Pneu-C-21 in public health programs ((7)), NACI sought to update recommendations on the use of pneumococcal vaccines in adults as part of its mandate. Economic evidence was determined to be a necessary component to inform the development of the vaccine guidance.

In support of NACI’s workplan ((8)), Canada’s Drug Agency (CDA; formerly Canadian Agency for Drugs and Technologies in Health) conducted a systematic review on the cost-effectiveness of pneumococcal conjugate vaccines in adults at high risk for pneumococcal disease (PD) aged 18 to 64 years ((9)). The systematic review generally found that Pneu-C-13, alone or in combination with Pneu-P-23, and Pneu-C-20 may be cost-effective compared to no vaccination at a threshold of $50,000/quality-adjusted life year (QALY) gained in populations at higher risk of IPD ((9)). Pneu-C-15 used in combination with Pneu-P-23 was unlikely to be cost effective at commonly used thresholds in high-risk adults. None of the included economic evaluations included Pneu-C-21 as an intervention or comparator.

The CDA systematic review focused on the question of whether pneumococcal conjugate vaccines are a cost-effective intervention in adults less than 65 years of age at risk for PD. We conducted a separate systematic review to address the policy question of whether Pneu-C-21 is cost-effective for preventing PD in adults aged 18 years and older. The aim of this review was to identify any more recently published studies and include all adults, including those aged 65 years and older.

## Methods

Our systematic review was informed by NACI’s Guidelines for Systematic Reviews on Economic Evaluations of Vaccination Programs ((10)). We conducted a literature search of EBM Reviews; Cochrane Central Register of Controlled Trials, EconLit, Embase, International Pharmaceutical Abstracts, Ovid MEDLINE and Scopus. In addition, we searched the websites of National Immunization Technical Advisory Groups, including the Joint Committee on Immunisation (United Kingdom), the Advisory Committee on Immunization Practices (ACIP; United States), Standing Committee on Immunization (Germany) and Australian Technical Advisory Group on Immunisation (Australia). Our search was limited to literature published in English and French from 2019 onward. The search strategy was developed in consultation with and validated by a librarian at the Health Canada Library. It is available directly from the authors as Supplemental material (see **Appendix** for more information). The search was completed on July 3, 2024.

Full texts were identified, retrieved and screened against our inclusion criteria by two reviewers (**Table 1**). Our inclusion criteria ensured included studies were full economic evaluations with Pneu-C-21 as the intervention. A Preferred Reporting Items for Systematic Reviews and Meta-Analyses (PRISMA) diagram ((11)) that details this process was developed.

**Table 1:** Policy question and inclusion criteria

Inclusion criteria	Description
Population	Adults aged 18 years and older
Intervention	21-valent pneumococcal conjugate vaccine (Pneu-C-21; V116)
Comparators	Any (i.e., placebo, no intervention, other pneumococcal vaccines)
Outcomes	QALYs, DALYs, incremental costs, incremental cost-effectiveness ratios (cost per QALY gained or incremental cost per event or event avoided), net monetary benefit, net health benefit
Study designs	Full economic evaluations (e.g., cost-utility analyses, cost-effectiveness analyses, cost-benefit analyses)^a^

Abbreviations: DALY, disability-adjusted life years; Pneu-C-21, 21-valent pneumococcal conjugate vaccine; QALY, quality-adjusted life year

^a^ Studies with only abstracts available were excluded

We extracted study characteristics, methods, findings and funding sources from evaluations that met our inclusion criteria. To ensure our findings were informative for NACI’s decision-making, we focused our review on health outcomes and costs for vaccination strategies that were under consideration (i.e., currently recommended strategies as comparators) (Appendix, Table S1). Costs were converted to 2023 Canadian dollars (CAD) using the Organisation for Economic and Co-operation and Development (OECD) purchasing parity rates ((12)) and the Bank of Canada’s inflation calculator ((13)). Our main outcome of interest was the incremental cost-effectiveness ratio (ICER). When comparators from a study were not aligned with NACI’s policy questions, we calculated ICERs using the relevant comparator based on the costs and QALYs provided in the published study. Included economic evaluations were critically appraised by one reviewer using the Joanna Briggs Institute Critical Appraisal Checklist for Economic Evaluations (JBI Checklist) ((14)). To complement the JBI Checklist, we also appraised included studies against three questions from World Health Organization for standardization of economic evaluations of immunization programmes ((10,15)). To assess the generalizability of the included studies (i.e., JBI Checklist item 11: “Are the results generalizable to the setting of interest in the review?”), we considered guidance from Heyland *et al.* ((16)).

## Results

**Figure 1:**
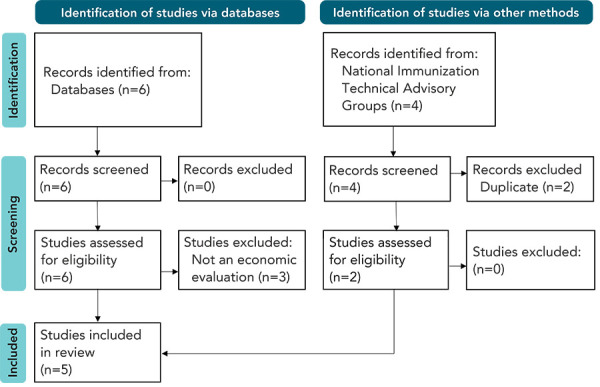
Preferred Reporting Items for Systematic Reviews and Meta-Analyses (PRISMA) diagram^a^ ^a^ Preferred Reporting Items for Systematic Reviews and Meta-Analyses (PRISMA) diagram ((11)) detailing the search and screening process used to select included economic evaluations on the use of a 21-valent pneumococcal conjugate vaccine (Pneu-C-21) in adults

Ten publications were identified in our search and five were included in our systematic review (**Figure 1**). Three economic evaluations were included from the peer-reviewed literature ((17–19)). Results from three economic evaluations were summarized and presented to ACIP ((20)), including a model by Altawalbeh *et al.* ((17)) that was also identified in the peer-reviewed literature. For clarity, the models summarised to ACIP are referred to by the names of the model authors (i.e., Altawalbeh *et al.*, 2024 ((17)); Owusu-Edusei *et al.*, 2024 ((21)); Stoecker, 2024 ((22))). One of the evaluations presented to ACIP was an industry-funded model by Merck ((21)).

All five economic evaluations used static cohort models to inform their cost-utility analyses (**Table 2**). Four evaluations were conducted in the United States ((17,19,21,22)) and one was conducted in the Netherlands ((18)). Three economic evaluations were conducted from the societal perspective ((18,21,22)), one was conducted from the health system perspective ((19)) and one included findings from both the societal and health system perspectives ((17)). The four studies conducted in the United States used a 3% discount rate ((17,19,21,22)) and de Boer *et al*. used a 4% discount rate for costs and a 1.5% discount rate for QALYs ((18)). The three peer-reviewed studies met nine out of 11 of the JBI Checklist criteria and were high quality (**Table 3**). The two models only presented at ACIP met between three and six of the 11 JBI Checklist criteria. Only one study ((18)) thoroughly discussed the strengths and weaknesses of their model in relation to pneumococcal transmission dynamics.

**Table 2:** Summary of included economic evaluations

Economic evaluations	Altawalbeh *et al.*, 2024 ((17))	de Boer *et al.*, 2024 ((18))	Owusu-Edusei *et al.*, 2024 ((21))	Stoecker, 2024 ((22))	Wateska *et al.*, 2023 ((19))
Country	United States	Netherlands	United States	United States	United States
Perspective	Health system and societal	Societal	Societal	Societal	Health system
Modelling approach	Static cohort model	Static cohort model	Static cohort model	Static multi-cohort model	Static cohort model
Inclusion of indirect effects/serotype replacement from pediatric vaccination	Indirect effects only	Indirect effects and serotype replacement	Indirect effects only	Indirect effects only	Indirect effects only
Time horizon	Lifetime	15 years	100 years	Varies	Lifetime
Discount rate	3%	4% for costs and 1.5% for QALYs	Assumed 3%	3%	3%
Study population	Adults aged >50 years and older and high risk adults younger than 50 years; stratified by race	Adults aged 60 years and older	Adults aged 19 years and older	Adults aged 19 years and older	Adults aged 65 years and older; stratified by race
Comparators	Pneu-C-20, Pneu-C-15+Pneu-P-23, no vaccination	Pneu-C-20, Pneu-C-15+Pneu-P-23, Pneu-C-15, no vaccination	Pneu-C-20	Pneu-C-20	Pneu-C-20, Pneu-C-15+Pneu-P-23, no vaccination
Price per dose	2019 USD Pneu-C-21: $333.00 Pneu-C-20: $249.00 Pneu-C-15: $216.09 Pneu-P-23: $117.08	2021 EUR Pneu-C-21: €82.17 Pneu-C-20: €82.17 Pneu-C-15: €74.73 Pneu-P-23: €25.94	2023 USD Pneu-C-21: $287 Pneu-C-20: $261	2023 USD Pneu-C-21: $319.43 Pneu-C-20: $288.66	2019 USD Pneu-C-21: $333.00 Pneu-C-20: $249.00 Pneu-C-15: $216.09 Pneu-P-23: $117.08
Price per dose (2023 CAD)^a^	Pneu-C-21: $466 Pneu-C-20: $349 Pneu-C-15: $303 Pneu-P-23: $164	Pneu-C-21: $148 Pneu-C-20: $148 Pneu-C-15: $135 Pneu-P-23: $47	Pneu-C-21: $333 Pneu-C-20: $303	Pneu-C-21: $371 Pneu-C-20: $335	Pneu-C-21: $466 Pneu-C-20: $349 Pneu-C-15: $303 Pneu-P-23: $164
Funding	National Institute of Allergy and Infectious Diseases	Netherlands Ministry of Health, Welfare and Sport	Merck industry model	None listed	None listed

Abbreviations: CAD, Canadian dollar; EUR, Euro; Pneu-C-15, 15-valent pneumococcal conjugate vaccine; Pneu-C-20, 20-valent pneumococcal conjugate vaccine; Pneu-C-21, 21-valent pneumococcal conjugate vaccine; Pneu-C-23, 23-valent pneumococcal polysaccharide vaccine; QALY, quality-adjusted life years; USD, United States dollar

^a^ Costs were converted to 2023 CAD using the Organisation for Economic and Co-operation and Development (OECD) purchasing parity rates ((12)) and Bank of Canada’s inflation calculator (consumer price index)

**Table 3:** Quality appraisal^a^ of included economic evaluations ((14,15))

Study (reference)	Joanna Briggs Institute checklist											WHO checklist		
	1	2	3	4	5	6	7	8	9	10	11	12	13	14
Altawalbeh *et al.*, 2024 ((17))	Yes	Yes	Yes	Yes	Yes	Yes	Yes	Yes	Yes	No	No	Yes	Unclear	Unclear
de Boer *et al.*, 2024 ((18))	Yes	Yes	Yes	Yes	Yes	Yes	Yes	Yes	Yes	No	No	Yes	Yes	Unclear
Owusu-Edusei *et al.*, 2024 ((21))^b^	Yes	Yes	Unclear	Unclear	Unclear	Unclear	Unclear	Yes	Unclear	No	No	Unclear	Unclear	Unclear
Stoecker, 2024 ((22))^b^	Yes	Yes	Unclear	Yes	Unclear	Unclear	Yes	Yes	Yes	No	No	Unclear	Unclear	Unclear
Wateska *et al.*, 2023 ((19))	Yes	Yes	Yes	Yes	Yes	Yes	Yes	Yes	Yes	No	No	Yes	Unclear	Unclear

Abbreviation: WHO, World Health Organization

^a^ Questions: 1) Is there a well-defined question?; 2) Is there a comprehensive description of alternatives?; 3) Are all important and relative costs and outcomes for each alternative identified?; 4) Has clinical effectiveness been established?; 5) Are costs and outcomes measured accurately?; 6) Are costs and outcomes valued credibly?; 7) Are costs and outcomes adjusted for differential timing?; 8) Is there an incremental analysis of costs and consequences?; 9) Were sensitivity analyses conducted to investigate uncertainty in estimates of cost or consequences?; 10) Do study results include all issues of concern to users?; 11) Are the results generalizable to the setting of interest in the review?; 12) Are the model structure and implicit or explicit assumptions clearly described?; 13) Is the model type (static, dynamic or stochastic) clearly stated and justified in light of likely changes to the force of infection and the role of chance in the transmission process? Have the model’s strengths and weaknesses been discussed?; 14) Has the model been validated? If so, has it been validated in as many facets of validation as possible?

^b^ Study methods and findings were extracted from slides presented to the American Committee on Immunization Practices (ACIP) and limited details were available

Each of the included economic evaluations assumed different age-specific serotype distributions of PD cases. However, serotypes included in Pneu-C-21 caused more IPD cases than serotypes included in Pneu-C-20 in all of the economic evaluations (Appendix, Figure S1). Detailed assumptions on the assumed impact of paediatric pneumococcal conjugate vaccination on PD in adults due to indirect effects are shown in Appendix, Table S2. Each economic evaluation included indirect effects from pediatric vaccination in primary ((18,22)) or sensitivity analyses ((17,19,21)).

**Figure 2:**
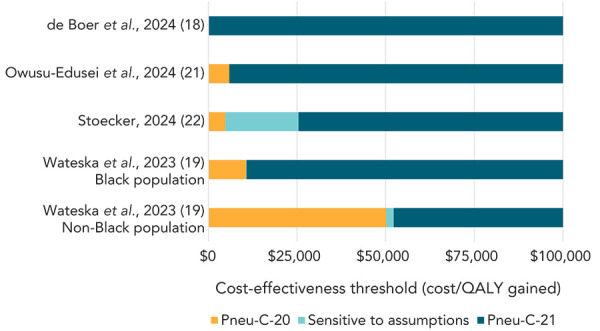
Preferred pneumococcal vaccination strategy in adults aged 65 years at cost-effectiveness thresholds ranging from $0/QALY gained to $100,000/QALY gained Abbreviations: Pneu-C-20, 20-valent pneumococcal conjugate vaccine; Pneu-C-21, 21-valent pneumococcal conjugate vaccine; QALY, quality-adjusted life years

Four of the economic evaluations included vaccinating all individuals aged 65 years with Pneu-C-20 as a comparator (Appendix, Table S3). Pneu-C-21 was the optimal vaccination strategy at a cost-effectiveness threshold of $50,000/QALY gained in this population, when compared to Pneu-C-20, in the majority of included studies (**Figure 2**). In the analysis by de Boer *et al.* ((18)), Pneu-C-20 was dominated by Pneu-C-21, meaning that Pneu-C-21 was both less costly and more effective than Pneu-C-20. Incremental cost-effectiveness ratios ranged from $4,793/QALY gained ((22)) to $52,265/QALY gained ((19)) when comparing Pneu-C-21 to Pneu-C-20 in the other economic evaluations (Figure 2; Appendix, Table S3).

**Figure 3:**
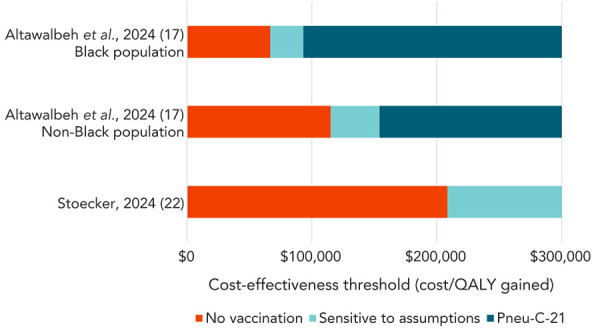
Preferred pneumococcal vaccination strategy in adults aged 50 years at cost-effectiveness thresholds ranging from $0/QALY gained to $300,000/QALY gained Abbreviations: Pneu-C-21, 21-valent pneumococcal conjugate vaccine; QALY, quality-adjusted life years

Two of the included economic evaluations compared the cost-effectiveness of vaccinating adults aged 50 years with Pneu-C-21 compared to no vaccination (Appendix, Table S3). Altawalbeh *et al.* ((17)) compared vaccinating Black and non-Black adults at the age of 50 years with Pneu-C-21 to no vaccination. In contrast, Stoecker ((22)) compared a strategy of vaccinating adults at the ages of 50 years and 65 years with Pneu-C-21 to a strategy of vaccinating adults at only the age of 65 years with Pneu-C-21, effectively comparing Pneu-C-21 at the age of 50 years to no vaccination in a population receiving Pneu-C-21 at the age of 65 years. Incremental cost-effectiveness ratios ranged from $66,706/QALY gained to $313,121/QALY gained ((17,22)), with the former reflecting the cost-effectiveness of vaccinating Black population members (**Figure 3**).

One economic evaluation compared the cost-effectiveness of vaccinating adults younger than 50 years of age at high risk of PD (Appendix, Table S3). In a cohort of adults aged 42 years living with immunocompromising conditions, including HIV, cancer, organ transplants and dialysis, Pneu-C-20 was dominated by Pneu-C-21 ((22)).

Catch-up vaccination was examined by Owusu-Edusei *et al.* ((21)) and Stoecker ((22)) in a range of age and risk groups (Appendix, Table S3). A catch-up dose of Pneu-C-21 between one and five years after a dose of Pneu-C-20 was never cost-effective at commonly used thresholds, with ICERs ranging from $239,128/QALY gained ((22)) to $594,229/QALY gained ((21)).

## Discussion

Our review identified five economic evaluations that assessed the cost-effectiveness of using Pneu-C-21 in adults. Three economic evaluations were summarized from the peer-reviewed literature ((17–19)) and two were summarized from ACIP presentations ((21,22)). In economic evaluations that included the strategy of vaccinating adults aged 65 years and older with Pneu-C-21 compared to Pneu-C-20, ICERs were around or below $50,000/QALY gained ((18,19,21,22)). A strategy of vaccinating adults aged 50 to 64 years with Pneu-C-21 compared to no vaccination had ICERs over $65,000/QALY gained, with the highest estimate being over $300,000/QALY gained ((17,22)). In adults younger than 50 years of age, a strategy with Pneu-C-21 dominated Pneu-C-20 in adults with immunocompromising conditions, but no vaccination dominated Pneu-C-21 in a strategy of vaccinating all adults (regardless of the presence of a chronic medical or immunocompromising condition) ((22)). Incremental cost-effectiveness ratios for a catch-up dose of Pneu-C-21 after vaccination with Pneu-C-20 were over $230,000/QALY gained ((21,22)). In the two studies that presented race-stratified results ((17,19)), ICERs were lower in the Black population compared to the non-Black population, primarily due to a higher risk of PD.

Although none of the included economic evaluations were conducted in Canada, they all employed cost-utility models, with health outcomes expressed as QALYs, which aligns with NACI’s guidelines for economic evaluations ((23)). Vaccine prices used in the economic evaluations conducted in the United States are higher than vaccine prices expected in Canada. An analysis commissioned by the United States Department of Health and Human Services and conducted by the RAND Corporation found that drug prices in Canada were, on average, 56% lower than those in the United States ((24)). Findings were especially sensitive to vaccine price assumptions when the comparator was no vaccination. In Canada, the recommended discount rate of future (i.e., beyond one year) costs and QALYs is 1.5% ((23)); with the exception of the discount rate of QALYs used by de Boer *et al.* ((18)), discount rates were greater than recommended by NACI’s guidelines ((17–19,21,22)). Altawalbeh *et al.* ((17)) was the only economic evaluation to present results from both the health system and societal perspective. The model by Owusu-Edusei *et al.* ((21)) is an industry-led model by Merck, the manufacturer of Pneu-C-21 ((5)). Finally, many of the vaccination strategies included in the economic evaluations were not relevant to current vaccine recommendations for adults living in Canada.

## Limitations

The results of the included economic evaluations were sensitive to key assumptions. First, the incidence of PD caused by vaccine-type and non-vaccine-type serotypes differs by region and over time, and the impact of the COVID-19 pandemic on PD dynamics is still unknown ((19)). Because higher valency pneumococcal conjugate vaccines for children are new, assumptions of the potential impact of indirect effects from pediatric vaccination with Pneu-C-15 or Pneu-C-20 were necessary ((18,20,22)). In a multi-model comparison, Leidner ((20)) identified the presence of indirect effects from pediatric vaccination, the PD case fatality rate, the prevalence and severity of long-term post-PD disability, productivity losses and vaccine price as key assumptions and parameters that impacted model findings. Altawalbeh *et al.* ((17)) and Wateska *et al.* ((19)) highlighted uncertainties surrounding vaccine price and vaccine effectiveness.

Additional limitations include the difficulty of assessing the quality of the economic evaluations presented to ACIP (because only presentation materials were available), the use of static models and assumptions regarding serotype replacement. To date, pneumococcal conjugate vaccines have been effective against *S. pneumoniae* colonization and their use has resulted in indirect (herd) effects. Dynamic transmission models are better equipped to capture the population level impact of pneumococcal conjugate vaccination strategies ((25)). Finally, with the exception of the model by de Boer *et al.* ((18)), none of the models included serotype replacement ((17,19,21,22)). Following the introduction of Pneu-C-13 in the routine pediatric vaccination schedule in Canada, serotype replacement resulted in a rise in IPD caused by the serotypes not included in the vaccine ((26,27)).

## Conclusion

Our systematic review of economic evaluations assessing the cost-effectiveness of Pneu-C-21 in adults to support guidance on its use in adults living in Canada adults suggests that it may be a cost-effective intervention compared to current recommendations in some populations. However, to best understand the potential cost-effectiveness of the use of Pneu-C-21 in adults living in Canada, a *de novo* economic evaluation that better reflects the Canadian context is required.

## Appendix

Additional data is available upon request to the author: alison.simmons@mail.utoronto.ca

EBM Reviews: Cochrane Central Register of Controlled Trials

EBM Reviews: EconLit

EBM Reviews: Embase

EBM Reviews: International Pharmaceutical Abstracts

EBM Reviews: Ovid MEDLINE(R) ALL

EBM Reviews: SCOPUS

Table S1: Current pneumococcal vaccination strategies for adults in Canada

Figure S1: Assumed age-specific pneumococcal serotype distributions in included economic evaluations

Table S2: Summary of assumptions relating to indirect effects and serotype replacement from pediatric pneumococcal conjugate vaccination in included economic evaluations

Table S3: Incremental cost-effectiveness ratios (ICERs) from selected strategies
